# Supplementary material for: Impaired host shutoff is a fitness cost associated with baloxavir marboxil resistance mutations in influenza A virus PA/PA-X nuclease domain
Source: PLoS Pathog. 2026 Feb 9;22(2):e1013550. doi: 10.1371/journal.ppat.1013550 (PMC12900429; doi:10.1371/journal.ppat.1013550)
Supplement: S1 Fig — (A) Sources of PA-X sequences and their amino acid identity. (B) BLASTP alignment of PA-X proteins from influenza A virus strains shown in panel A. PR8, CA07, SW13, TX24, and BC24 PA-X proteins are featured in this study; WS22 (H1N1) and DC23 (H3N2) are the recommended vaccine strains for the 2024–2025 season representing currently circulating viruses and are included for comparison. Positions of the isoleucine 38 and aspartate 108 in the nuclease active site are highlighted in yellow and red, respectively. Phenylalanine 191 marking the frame shift site and the beginning of the X-ORF is highlighted in grey. (PDF) [file ppat.1013550.s001.pdf]

**A**

| Label | Strain | Description | Accession | Identity |
|---|---|---|---|---|
| PR8: | A/Puerto Rico/8/34(H1N1) | Lab adapted strain | AF389117 | - |
| CA07: | A/California/7/09(H1N1) | 2009 pandemic H1N1 | MN596848 | 92.67% |
| SW13: | A/Switzerland/9715293/13(H3N2) | H3N2 from 2013 | OQ350031 | 93.65% |
| BC24: | A/BC/PHL-2032/24(H5N1) | Human H5N1 isolate | PV612033 | 94.05% |
| TX24: | A/Bovine/Texas/24-029328-01/24(H5N1) | Bovine H5N1 | PP599464 | 91.67% |
| WS22: | A/Wisconsin/67/22(H1N1) | Seasonal H1N1 | OQ203981 | 90.52% |
| DC23: | A/District of Columbia/27/23(H3N2) | Seasonal H3N2 | OQ665199 | 93.65% |

**B**

```
PR8   (H1N1)  1     MEDFVRQCFN PMIVELAEKT MKEYGEDLKI ETNKFAAICT HLEVCFMYSD FHFINEQGES  60
DC23  (H3N2)  1     .......... .........A .......... .......... .......... ..........  60
SW13  (H3N2)  1     .......... .........A .......... .......... .......... ..........  60
BC24  (H5N1)  1     .......... .........A .......P.. .......... .......... ....D.R...  60
TX24  (H5N1)  1     .......... .........A .......P.. .......... .......... ....D.R...  60
CA07  (H1N1)  1     .......... .......G.A .......P.. .......... .......... ....D.R...  60
WS22  (H1N1)  1     .......... .........A .......P.V .......... .......... ....D.R...  60

PR8   (H1N1)  61    IIVELGDPNA LLKHRFEIIE GRDRTMAWTV VNSICNTTGA EKPKFLPDLY DYKENRFIEI  120
DC23  (H3N2)  61    .V...D.... .......... .......... .......... .......... ..........  120
SW13  (H3N2)  61    .V...D.... .......... .......... .......... G......... ..........  120
BC24  (H5N1)  61    ....S..... .......... .......... .........V .......... ..........  120
TX24  (H5N1)  61    M...S..... .......... ....A..... .........V .......... ..R.......  120
CA07  (H1N1)  61    ....S..... .......... ....I..... .........V .......... ..........  120
WS22  (H1N1)  61    M.L.S..... .......... ....I..... .........I .......... ..........  120

PR8   (H1N1)  121   GVTRREVHIY YLEKANKIKS EKTHIHIFSF TGEEMATKAD YTLDEESRAR IKTRLFTIRQ  180
DC23  (H3N2)  121   .......... .......... .......... .......R.. .......... ..........  180
SW13  (H3N2)  121   .......... .......... .N........ .......... .......... ..........  180
BC24  (H5N1)  121   .......... .......... .......... .......... .......... ..........  180
TX24  (H5N1)  121   .......... .......... .......... .......... .......... ..........  180
CA07  (H1N1)  121   .......... .......... .......... .......... .......... ..........  180
WS22  (H1N1)  121   .......... .......... .......... .......... .......... ..........  180

PR8   (H1N1)  181   EMASRGLWDS FVSPREEKRQ LKKGLKSQEQ CASLPTKVSR RTSPALKILE PMWMDSNRTA  240
DC23  (H3N2)  181   ...N...... ....K.A.K. ...N....GL ..G......H ......R... ..........  240
SW13  (H3N2)  181   ...N...... ....K.A.K. ...N.....L ..G......H ......R... ..........  240
BC24  (H5N1)  181   .......... ....K.A... ...D.....P ..G......H .......T.. ..........  240
TX24  (H5N1)  181   .......... ..N...A... ...D.....P ..G.....FH .......T.. ..........  240
CA07  (H1N1)  181   .....S.... ....K.A... ...N.RL..L .........H .......T.. ..          232
WS22  (H1N1)  181   .....S.... ....K.A.K. ...S..L..L ..G......H Q......TS. ..          232

PR8   (H1N1)  241   TLRASCLKCP KK  252
DC23  (H3N2)  241   A....F.... ..  252
SW13  (H3N2)  241   A....F.... ..  252
BC24  (H5N1)  241   A....F.... ..  252
TX24  (H5N1)  241   A....F...Q .R  252
```

**Figure S1. PA-X protein alignment.** (A) Sources of PA-X sequences and their amino acid identity. (B) BLASTP alignment of PA-X proteins from influenza A virus strains shown in panel A. PR8, CA07, SW13, TX24, and BC24 PA-X proteins are featured in this study; WS22 (H1N1) and DC23 (H3N2) are the recommended vaccine strains for the 2024-2025 season representing currently circulating viruses and are included for comparison. Positions of the isoleucine 38 and aspartate 108 in the nuclease active site are highlighted in yellow and red, respectively. Phenylalanine 191 marking the frame shift site and the beginning of the X-ORF is highlighted in grey.
