# Supplementary material for: Impaired host shutoff is a fitness cost associated with baloxavir marboxil resistance mutations in influenza A virus PA/PA-X nuclease domain
Source: PLoS Pathog. 2026 Feb 9;22(2):e1013550. doi: 10.1371/journal.ppat.1013550 (PMC12900429; doi:10.1371/journal.ppat.1013550)
Supplement: S2 Fig — Immunofluorescence microscopy image of 293A cells that were treated with 500 µM sodium arsenite for 50 min (+As) or left untreated (-). Insets highlight representative areas of the cytoplasm with sodium arsenite-induced stress granules showing co-localization of PABPC1 (red) and G3BP1 (green). Nuclei are stained with Hoechst dye (teal). Scale bar = 100 µm. (PDF) [file ppat.1013550.s002.pdf]

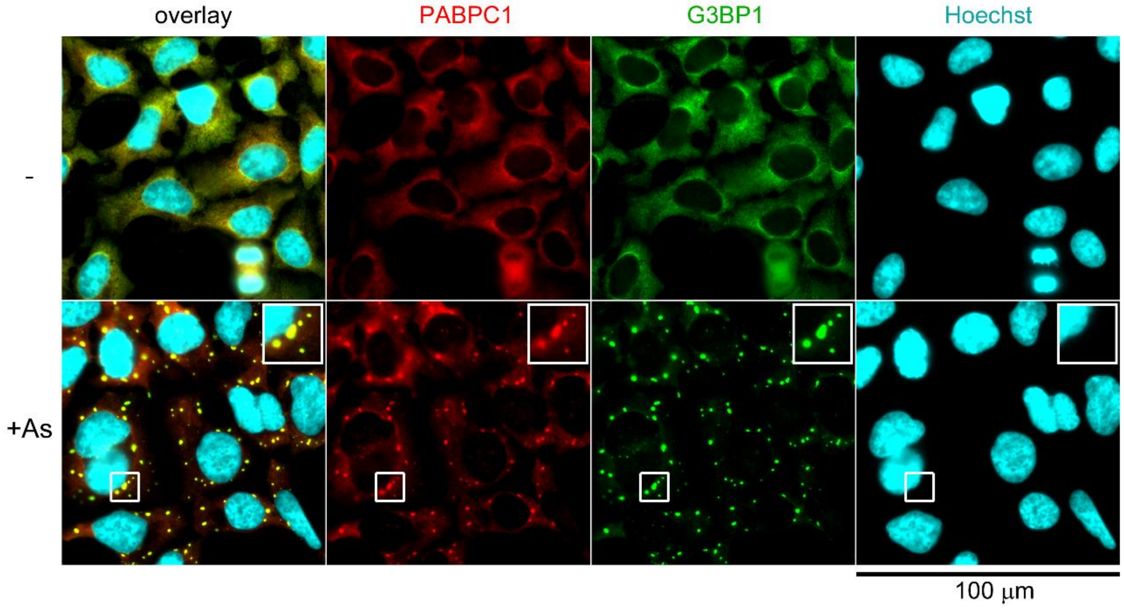

Figure S2. Co-localization of PABPC1 and G3BP1 in stress granules. Immunofluorescence microscopy image of 293A cells that were treated with 500 µM sodium arsenite for 50 min (+As) or left untreated (-). Insets highlight representative areas of the cytoplasm with sodium arsenite-induced stress granules showing co-localization of PABPC1 (red) and G3BP1 (green). Nuclei are stained with Hoechst dye (teal). Scale bar = 100 µm.
